# Dexmedetomidine ameliorates acute lung injury following orthotopic autologous liver transplantation in rats probably by inhibiting Toll-like receptor 4–nuclear factor kappa B signaling

**DOI:** 10.1186/s12967-015-0554-5

**Published:** 2015-06-13

**Authors:** Xinjin Chi, Xiaoxia Wei, Wanling Gao, Jianqiang Guan, Xiaofan Yu, Yiheng Wang, Xi Li, Jun Cai

**Affiliations:** Department of Anesthesiology, Third Affiliated Hospital, Sun Yat-sen University, Guangzhou, Guangdong 510630 China; Department of Anesthesiology, Fujian Provincial Hospital, Fuzhou, Fujian 350001 China; Department of Anesthesiology, First Affiliated Hospital, University of South China, Hengyang, Hunan 421001 China; Department of Thyroid and Breast Surgery, Third Affiliated Hospital, Sun Yat-sen University, Guangzhou, Guangdong 510630 China

**Keywords:** Liver transplantation, Acute lung injury, Toll-like receptor 4, Dexmedetomidine

## Abstract

**Background:**

To investigate whether pretreatment with dexmedetomidine (Dex) has a protective effect against acute lung injury (ALI) in an orthotopic autologous liver transplantation (OALT) rat model and to explore the mechanisms responsible for the protective effect of Dex against lung injury.

**Methods:**

Forty-eight rats underwent OALT and were randomly divided into six groups (n = 8 in each group) that received 10 µg/kg Dex, 50 µg/kg Dex, 50 µg/kg Dex + nonspecific α_2_-adrenergic receptor (AR) antagonist atipamezole, 50 µg/kg Dex + specific α_2B/C_-AR antagonist ARC-239, 50 µg/kg Dex + specific α_2A_-AR antagonist BRL-44408, or the same amount of normal saline. The sham rats (n = 8) underwent anesthesia induction, laparotomy, and separation of the portal vein without liver ischemia and reperfusion. Lung tissue sections were stained with hematoxylin and eosin (HE) to visualize the damage. The expression of Toll-like receptor 4 (TLR4) and the phospho-nuclear factor (NF)-κB p65 subunit as well as inflammatory cytokines was measured.

**Results:**

Rats exhibited increased histological lung injury scores and pulmonary edema following OALT. Pretreatment with 50 μg/kg Dex attenuated OALT-induced lung injury in rats, probably by inhibiting the activation of the TLR4–NF-κB signaling pathway. The protective effect of Dex could be blocked by atipamezole or BRL-44408, but not by ARC-239, suggesting these effects of Dex were mediated, at least in part, by the α_2A_-AR.

**Conclusions:**

Dex exerts protective effects against ALI following OALT, and this protection is associated with the suppression of TLR4–NF-κB signaling. Thus, pretreatment with Dex may be a useful method for reducing lung damage caused by liver transplantation.

## Background

Acute lung injury (ALI) is a common complication following liver transplantation, with an incidence between 34.2 and 77.8% [1, 2]. ALI is a progressive, devastating disease characterized by bilateral lung infiltration, hypoxemia refractory to oxygen therapy, and decreased lung compliance. Patients who develop ALI may further develop acute respiratory distress syndrome (ARDS), which has become one of the major causes of death after liver transplantation [3, 4]. Therefore, treatment strategies to protect against ALI after liver transplantation have been studied.

Toll-like receptor 4 (TLR4) is a member of the TLR family that plays a key role in both innate and adaptive immune responses. Triggering of the TLR pathway leads to activation of nuclear factor kappa B (NF-κB) and subsequent regulation of immune and inflammatory genes/factors, such as tumor necrosis factor (TNF)-α, interleukin (IL)-1, IL-6, and IL-8 [5–7]. Previous studies revealed increased expression of TLR4 in peripheral blood mononuclear cells and pro-inflammatory cytokines during the perioperative period of liver transplantation, indicating the involvement of TLR4 signaling in the development of ALI after liver transplantation [3, 8].

The alpha 2 adrenergic receptor (α_2_-AR) is a prototypical G protein-coupled receptor (GPCR). α_2_-AR mediates the physiological and pharmacological actions of catecholamines via G-proteins to a variety of effectors, including adenylyl cyclases and ion channels [9, 10]. The α2-AR subfamily includes three different subtypes (α_2A_, α_2B_, and α_2C_) in mammals, and they are found ubiquitously in vital organs and blood vessels [11, 12]. Dexmedetomidine (Dex) is a potent agonist for all three subtypes of human α_2_-ARs, having sedative, analgesic, and anti-sympathetic effects [11]. Dex has been shown to provide good perioperative hemodynamic stability and to have a protective effect on specific organs, including the heart, brain, and kidney, probably via decreasing cell apoptosis in a Bax/Bcl-2-related manner [13] and inhibiting pro-inflammatory cytokine release [14–16].

To date, few studies have examined the protective effects of Dex against perioperative ALI after liver transplantation. Our study aimed to investigate whether pretreatment with Dex resulted in a protective effect against ALI in an orthotopic autologous liver transplantation (OALT) rat model and to explore the underlying mechanisms of this protective effect.

## Methods

### Animals

Male Sprague–Dawley rats (aged 8–10 weeks, weighing 220–250 g) were purchased from the Medical Experimental Animal Center of Guangdong Province, China. The study was conducted in accordance with the Guide for the Care and Use of Laboratory Animals (National Institutes of Health, 1985) [17] and was approved by the local Ethical Committee of the Third Affiliated Hospital, Sun Yat-Sen University.

### Preparation of experimental animal models

The rats were kept under observation in a constant environment (room temperature 25–27°C) for 1 week prior to the start of the experiments. The OALT model was established in Sprague–Dawley rats as previously reported [18]. Briefly, under anesthesia, the falciform ligament of the liver of the rat was severed. The first hepatic portal was dissected, and the portal vein (PV) was liberated. The hepatic artery and biliary tract were liberated together based on their anatomic relationship. Vascular clamps were applied at the convergence of the inferior mesenteric, splenic veins, hepatic artery, supra hepatic vena cava (SVC), and inferior vena cava (IVC). The PV was punctured with a needle in preparation for reperfusion using pre-cooled Ringer lactate solution. Finally, the needle was extracted, and the PV, SVC, IVC, and hepatic artery were unclamped. All rats were sacrificed 8 h after liver reperfusion for collection of blood and lung samples.

### Grouping and drug treatment

Forty-eight rats subjected to OALT were randomly divided into six groups (n = 8 in each group) that received 10 µg/kg Dex (group D1), 50 µg/kg Dex (group D2), 50 µg/kg Dex + nonspecific α_2_-AR antagonist atipamezole (group B1), 50 µg/kg Dex + specific α_2B/C_-AR antagonist ARC-239 (group B2), 50 µg/kg Dex + specific α_2A_-AR antagonist BRL-44408 (group B3), or the same amount of normal saline (model group or group M) [19]. The rats were intraperitoneally injected with Dex at different doses 30 min before OALT, and 500 µg/kg atipamezole, 1.5 mg/kg BRL-44408, or 50 µg/kg ARC-239 40 min before OALT, according to the group designations. The dose selection of antagonists was based on the antagonists’ affinity and dose–effect relationship with Dex. The sham rats (n = 8; the sham group or group S) underwent anesthesia induction, laparotomy, and separation of the portal vein without liver ischemia and reperfusion.

### Histological analysis of lung tissues

Lung tissues were fixed, sectioned at 4 μm thickness, and stained with hematoxylin and eosin (HE). The sections were scored as previously reported, and the histological scoring parameters included edema of the alveoli, edema of the alveolar mesenchyme, intra-alveolar cell infiltration, alveolar hemorrhage, and atelectasis [18].

### Lung wet-to-dry (W/D) weight ratio

The wet (W) and dry (D) weights (after drying in ventilated oven at 80°C for 24 h) of the right middle lung lobes were measured. The lung W/D weight ratio was calculated by dividing the wet weight by the dry weight.

### Western blotting analysis of TLR4 expression

Protein samples were extracted from the lung tissues. Samples (20 μg protein) were loaded onto a 10% sodium dodecyl sulfate polyacrylamide gel electrophoresis (SDS-PAGE) gel and then transferred onto a polyvinylidene difluoride (PVDF) membrane. The membrane was blocked with 5% fat-free in Tris-buffered saline-Tween (TBS-T) blocking solution (containing 0.2% Tween-20, 20 mmol/L Tris–HCl, and 150 mmol/L NaCl, pH 7.14) at 37°C for 1 h, followed by incubation with primary antibody (1:1,000; Abcam, USA) at 37°C for 2 h. Membranes were washed with TBS solution containing 1% milk four times and then incubated at room temperature for 1 h with horseradish peroxidase (HRP)-conjugated secondary antibody (dilution 1:2,000, Santa Cruz Biotechnology, CA, USA). The bands on the membranes were visualized by an enhanced chemiluminescence (ECL) system. The density of each band was quantified by an image analyzer (Lab Works Software, CA, USA) and corrected by reference to the expression value for glyceraldehyde-3-phosphate dehydrogenase (GAPDH).

### Immunofluorescent assay and laser scanning confocal microscopy (LSCM)

Paraffin-embedded sections of lung tissue were dewaxed and rehydrated. Antigen retrieval was performed by heating in citrate buffer (pH = 6.0). Then, the sections were treated with 3% hydrogen peroxide (H_2_O_2_) for 10 min and incubated with a primary rabbit phospho-NF-κB p65 antibody (Cell Signaling Technology, MA, USA) at a dilution of 1:100, overnight at 4°C. The sections were washed with phosphate-buffered saline (PBS) and then further incubated with a fluorescein isothiocyanate (FITC)-conjugated goat anti-rabbit secondary antibody at a dilution of 1:100 (Life Technologies, USA) for 2 h at room temperature in the dark. The sections were counterstained with 4,6-diamidino-2-phenylindole (DAPI) to visualize nuclei. Images were captured by LSCM (Zeiss LSM 510 META, Jena, Germany).

### Enzyme-linked immunosorbent assay (ELISA)

The lung tissues were homogenized and centrifuged. The TNF-α and IL-1β levels in the lung tissues were measured using commercially available ELISA kits (Keygen Biotech, Nanjing, China), according to the instructions provided by the manufacturer.

### Detection of myeloperoxidase (MPO) activity

MPO activity, an indicator of polymorphonuclea (PMN) infiltration, was determined as previously described [20]. MPO activity was defined as the quantity of enzyme that degraded 1 mmol H_2_O_2_ at 37°C, and it was expressed as U/g wet tissue.

### Statistical analysis

All statistical analyses were performed using SPSS 13.0 (SPSS, Inc., IL, USA). Tests for normality and homogeneity of variances were performed. For normally distributed data, quantitative data are presented as mean ± standard deviation (SD). One-way analysis of variance (ANOVA) was used to test for differences among the seven groups. A statistical difference was considered when the P value was less than 0.05.

## Results

### Histological findings

**Figure 1:**
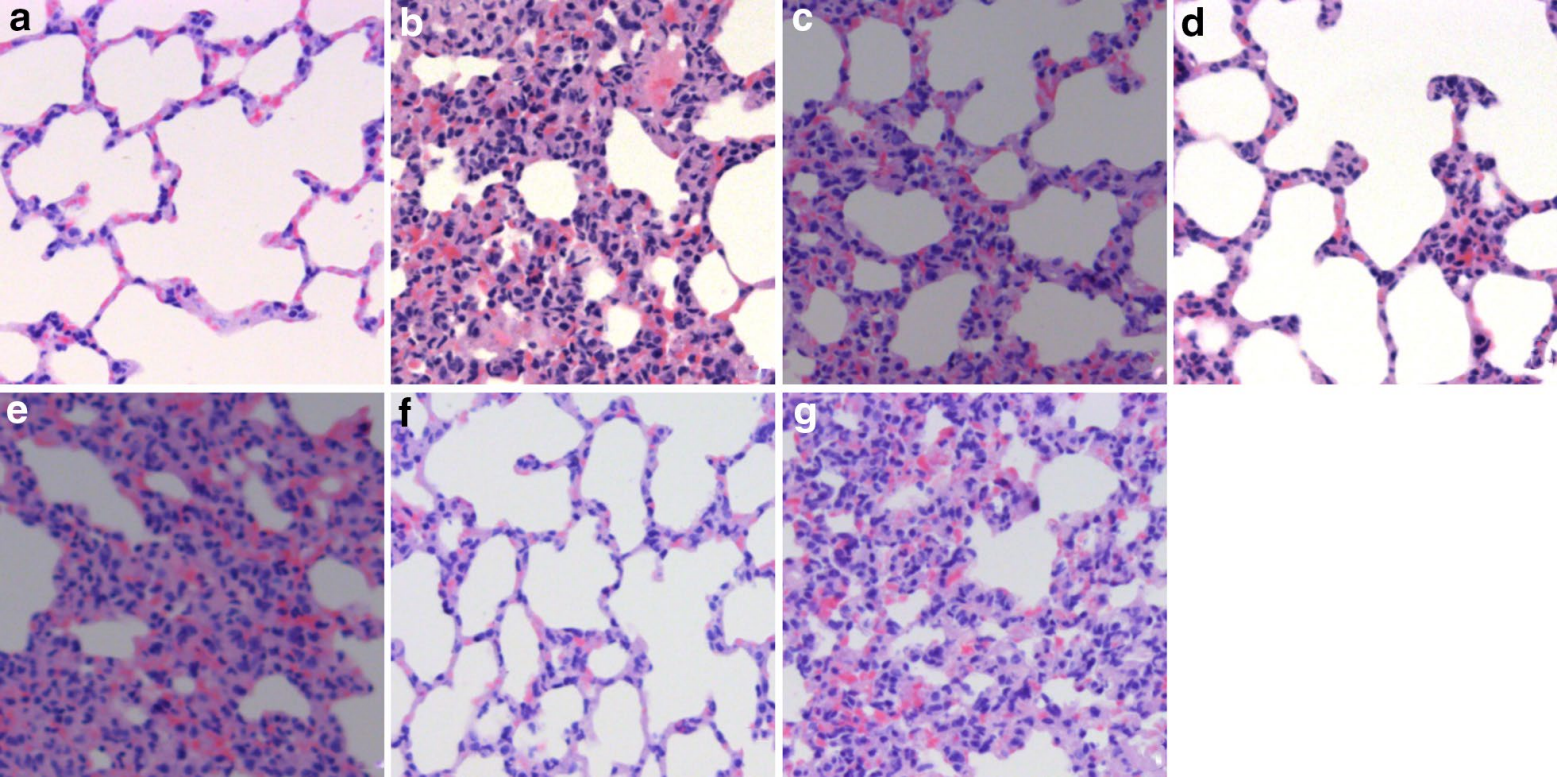
Pathological changes in lung tissue in each group. Images (magnification, ×200) of representative sections from rat lungs stained with hematoxylin and eosin after harvest from group S (**a**), group M (**b**), group D1 (**c**), group D2 (**d**), group B1 (**e**), group B2 (**f**), and group B3 (**g**).

**Figure 2:**
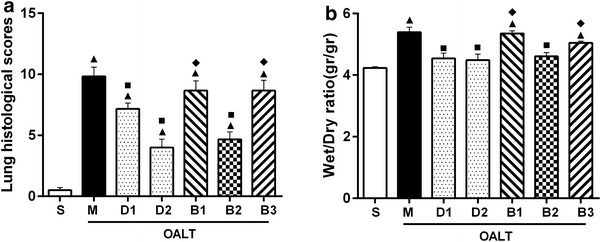
Pathological scores of lung injury (**a**) and lung wet-to-dry (W/D) weight ratio (**b**) in each group. The data are expressed as mean ± standard deviation (SD) (n = 8). ^▲^ *P* < 0.01 vs. group S; ^■^ *P* < 0.01 vs. group M; ^◆^ *P* < 0.01 vs. group D2.

Results obtained from light microscopy in the sham group (group S) showed complete structural integrity, clear alveolar space, and normal alveolar septum. In contrast, extensive pulmonary destruction, including massive inflammatory cell infiltration, obvious pulmonary hemorrhage, marked thickening of alveolar septum, and significant increase in the proportion of lung parenchyma, was observed in the group M. The rats in group M showed a higher pathological score than those in group S (*P* < 0.01). Dex pretreatment effectively reduced inflammatory cell infiltration and inhibited thickening of alveoli septum in lung tissue, and this effect showed a dose–effect relationship. Rats pretreated with 10 µg/kg or 50 µg/kg Dex had lower pathological scores than rats in group M (*P* < 0.01). Interestingly, the protective effect of Dex could be blocked by the nonspecific α_2_-AR antagonist atipamezole or α_2A_-AR antagonist BRL-44408 (*P* < 0.01, group B1 or B3 vs. group D2) but not by the α_2B/C_-AR antagonist ARC-239 (*P* > 0.05, group B2 vs. group D2; Figures 1, 2a).

### Lung W/D weight ratio

The lung W/D weight ratio was used as an index of water accumulation in the lung. The lung water content of the right middle lobes in rats increase significantly after OALT (*P* < 0.01, group M vs. group S). Pretreatment with 10 µg/kg or 50 µg/kg Dex remarkably reduced the water content of lung (*P* < 0.01, group D1 or D2 vs. group M). Treatment with atipamezole or BRL-44408, but not ARC-239, weakened the protective effect of Dex against pulmonary edema (*P* < 0.01, group B1 or B3 vs. group D2; Figure 2b).

### Expression of TLR4 and phospho-NF-κB p65 subunit in lung tissues

**Figure 3:**
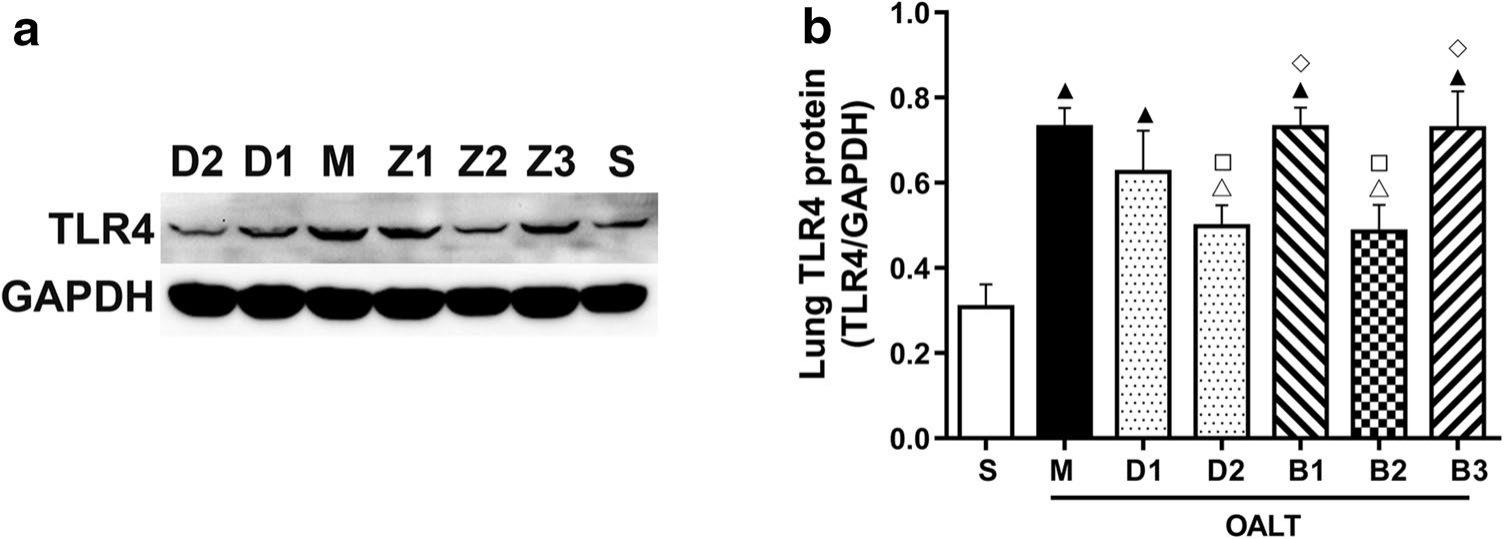
The expression of TLR4 protein in lung tissues of different groups. **a** The levels of TLR4 protein were determined by Western blotting analysis. **b** Quantification of the Western blotting data and correction by reference to the value of GAPDH expression. The data are expressed as mean ± SD (n = 8). ^▲^ *P* < 0.01; ^△^ *P* < 0.05 vs. group S; ^□^ *P* < 0.05 vs. group M; ^◇^ *P* < 0.05 vs. group D2.

The expression of TLR4 protein was measured by Western blotting. TLR4 protein expression was significantly enhanced in lung tissues following OALT (*P* < 0.01, group M vs. group S). Pretreatment with Dex at 50 µg/kg downregulated the expression of TLR4 protein (*P* < 0.01, group D2 vs. group M), and this effect was reversed by treatment with atipamezole or BRL-44408 (*P* < 0.05, group B1 or B3 vs. group D2), but not by ARC-239 (*P* > 0.05, group B2 vs. group D2; Figure 3).

**Figure 4:**
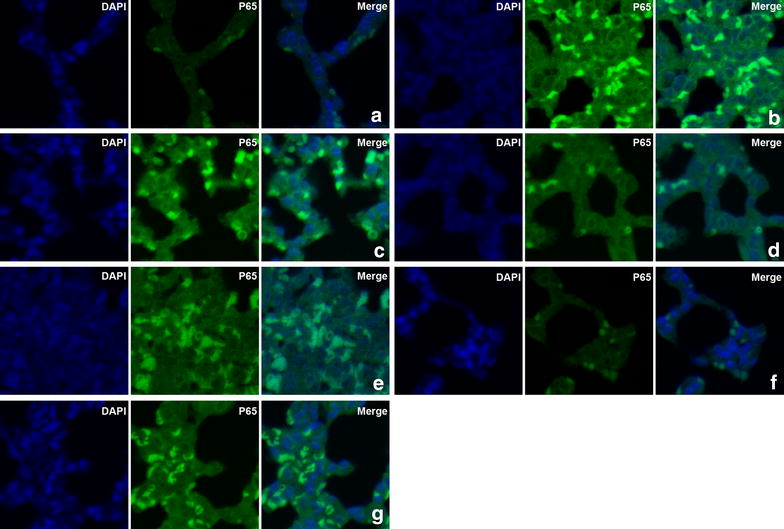
The expression of the phospho-NF-κB p65 subunit in lung tissues of different groups was detected by immunofluorescence staining under laser scanning confocal microscopy (magnification, ×400). **a** Group S; **b**, group M; **c**, group D1; **d**, group D2; **e**, group B1; **f**, group B2; and **g**, Group B3. Cells positive for phospho-NF-κB p65 were stained *green*, with the sections counterstained with 4,6-diamidino-2-phenylindole (DAPI) to visualize nuclei.

**Figure 5:**
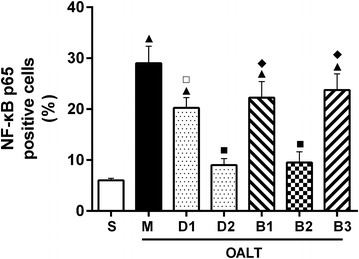
The proportion of phospho-NF-κB subunit p65 positive cells in lung tissues of different groups. The cells were counted under laser scanning confocal microscopy (magnification, ×400). The data are expressed as mean ± standard deviation (SD) (n = 8). ^▲^ *P* < 0.01; ^△^ *P* < 0.05 vs. group S; ^□^ *P* < 0.05 vs. group M; ^◇^ *P* < 0.05 vs. group D2.

Cells stained positively for phospho-NF-κB were observed and counted under LSCM (magnification, 400×). The expression of the phospho-NF-κB p65 subunit was significantly increased in rats after OALT (*P* < 0.01, group M vs. group S), suggesting an increased transport of activated NF-κB complexes into the nucleus. Pretreatment with 10 µg/kg or 50 µg/kg Dex down-regulated the elevated phospho-NF-κB p65 subunit expression after OALT (*P* < 0.01, group D1 or D2 vs. group M), indicating that Dex pretreatment might inhibit the activation and nuclear transport of NF-κB proteins. The effect of Dex on the phospho-NF-κB p65 subunit was diminished by treatment with atipamezole or BRL-44408 (*P* < 0.05, group B1 or B3 vs. group D2), but not ARC-239 (*P* > 0.05, group B2 vs. group D2; Figures 4, 5).

### TNF-α and IL-1β concentrations and MPO activity in lung tissues

**Figure 6:**
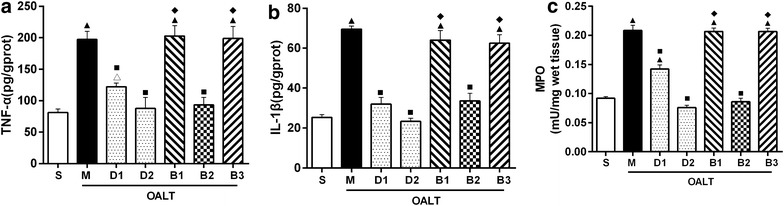
The concentrations of TNF-α (**a**) and IL-1β (**b**) and MPO activity (**c**) in lung tissues of different groups. The data are expressed as mean ± SD (n = 8). ^▲^ *P* < 0.01; ^△^ *P* < 0.05 vs. group S; ^■^ *P* < 0.01 vs. group M; ^◆^ *P* < 0.01 vs. group D2.

Production of pro-inflammatory factors in the lung is considered indicative of the severity of lung injury [21]. To evaluate the production of pro-inflammatory factors following OALT, TNF-α and IL-1β levels were examined by ELISA. The TNF-α and IL-1β levels in lung tissues were significantly higher in group M than in group S (*P* < 0.05). Pretreatment with Dex appeared to have a stronger effect on reducing the levels of TNF-α and IL-1β (*P* < 0.05, group D1 or D2 vs. group M). However, these effects of Dex could be almost completely blocked by treatment with atipamezole or BRL-44408 (*P* < 0.05, group B1 or B3 vs. group D2), but not by ARC-239 (*P* > 0.05, group B2 vs. group D2). The MPO activity in lung tissues was consistent with TNF-α and IL-1β levels and showed a similar trend among the groups (Figure 6).

## Discussion

This study investigated whether pretreatment with Dex has a protective effect against ALI in an OALT rat model. We found that Dex exerts protective effects against ALI following OALT, and this protection was mediated, at least in part, by the α_2A_-AR and was associated with the suppression of TLR4–NF-κB signaling.

In this study, we successfully established an OALT model according to a method used in previous studies [22]. This liver model can exactly imitate human pathophysiologic conditions of liver transplantation. Pathological changes in lung tissues following OALT included extensive pulmonary destruction such as massive inflammatory cell infiltration, obvious pulmonary hemorrhage, and marked alveolar septum thickening. Moreover, the pulmonary edema evaluated according to the W/D weight ratio was markedly increased after OALT as well. These results, taken together, provide definitive evidence for ALI in the OALT model. We chose 8 h after transplantation as the observation point based on our previous research, which indicated that rats present the most serious damage to the lung at 8 h after liver transplantation.

Several triggering conditions, including hemorrhagic shock, endotoxin, trauma, burn injury, and ischemia–reperfusion, contribute to ALI and exaggerate the inflammatory process of ALI [23–25]. NF-κB is an important nuclear transcription factor that upon activation, transfers into the nucleus, amplifying inflammatory responses by promoting production of pro-inflammatory cytokines such as TNF-α, IL-6, and IL-8 [26, 27]. TLR4 plays an important role in the pathogenesis of ALI via activation of NF-κB signaling [7, 8, 24]. In a lipopolysaccharide (LPS)-induced ALI model, the gene expression level of pulmonary cytokines, such as TNF-α, IL-1β, and IL-6, is significantly increased in wild-type mice compared with TLR4-deficient mice [28]. A study by Lorenz et al. found that TLR4-deficient murine macrophage cells are nonresponsive to stimulation by endotoxically active LPS of gram-negative bacteria, even under a high concentration [29]. Although direct blockade of TLR4 may potentially serve as an effective therapeutic strategy for acute pulmonary inflammation and ALI [30, 31], blockade of TLR4 must be applied with caution, because TLR4 blockade is not beneficial for patients with sepsis [32]. Our findings revealed that the TLR4–NF-κB signaling pathway is involved in the pathogenesis of ALI after liver transplantation, as evidenced by increased levels of TLR4 and phospho-NF-κB p65 subunit protein as well as proinflammatory cytokines TNF-α and IL-1β in the injured lung.

Dex has been reported to exert protective effects in various pulmonary conditions [15, 33]. Dex may directly suppress the immune response by modulating TLR4 expression, which results in inhibition of inflammatory cytokine production. A study by Want et al. demonstrated that Dex might exert its protective effects against hydrogen sulfide-induced ALI through down-regulation of matrix metalloproteinase (MMP)-2 and MMP-9 expression [34]. It was also reported that pretreatment with Dex significantly reduces the lung injury caused by ischemia–reperfusion injury [15]. Our study found that pretreatment with Dex could cause a significant decrease in lung pathological scores and the lung W/D weight ratio, suggesting a protective effect of Dex on lung tissue. NF-κB activation was attenuated in rats treated with Dex, which was consistent with the changes in the expression of TLR4 as well as pro-inflammatory cytokines TNF-α and IL-1β in the lung tissue following OALT. Yang et al. [33] reported that Dex at the dose of 5.0 µg/kg per h significantly attenuated the effects of ventilator-induced lung injury, which was mediated by the α_2_-AR.

Human and rat kidneys are reported to contain all three types of α_2_-ARs [35]. In this study, we used different α_2_-AR antagonists to elucidate the contribution of these α_2_-AR subtypes to the effects of Dex. Interestingly, the protective effects of Dex could be blocked by the nonspecific α_2_-AR antagonist atipamezole or α_2A_-AR antagonist BRL-44408, but not by the α_2B_-AR antagonist ARC-239, suggesting these effects of Dex were mediated, at least in part, by the α_2A_-AR. Our results are consistent with previous findings that Dex provides potent neuroprotection mediated via the α_2A_-AR subtype in a model of perinatal excitotoxic brain injury [36]. The exact mechanism by which Dex exerts its protective effects via the α_2A_-AR requires further investigation.

## Conclusions

Dex exerts protective effects against ALI following OALT, and this protection is associated with the suppression of TLR4–NF-κB signaling, which may explain the inhibitory effect of Dex on the production of pro-inflammatory factors TNF-α and IL-1β. These effects of Dex were mediated by the α_2A_-AR. Thus, pretreatment with Dex may be a useful method for reducing the lung damage caused by liver transplantation.
